# Evaluating the effect of garlic extract on serum inflammatory markers of peritoneal dialysis patients: a randomized double-blind clinical trial study

**DOI:** 10.1186/s12882-019-1204-6

**Published:** 2019-01-25

**Authors:** Elham Zare, Amirhesam Alirezaei, Mahmood Bakhtiyari, Asieh Mansouri

**Affiliations:** 1 Department of Internal Medicine, ShahidBeheshti University of Medical Sciences, Tehran, Iran; 2 Clinical Research Developement Center at ShahidModarres Hospital, Department of Nephrology, ShahidBeheshti University of Medical Sciences, Tehran, Iran; 3 Non-communicable Diseases research center, Alborz University of Medical Sciences, Karaj, Iran; 4 Department of Community Medicine, School of Medicine, Alborz University of Medical Sciences, Karaj, Iran; 5 Hypertension Research Center, Cardiovascular Research Institute, Isfahan University of Medical Sciences, Isfahan, Iran

**Keywords:** Garlic extract, Peritoneal dialysis (PD), Serum inflammatory markers

## Abstract

**Background:**

Garlic can be considered as a useful natural herb in inhibition of inflammation. The aim of this study was to assess the effectiveness of garlic extract in lowering inflammatory markers in peritoneal dialysis (PD) patients.

**Methods:**

In this parallel-designed double blind randomized clinical trial, 42 PD patients at the Shafa dialysis center, Tehran in 2017 were included. The primary outcome in this study was systemic inflammation which was evaluated by measuring the concentrations of IL-6 and CRP and ESR in serum.

**Results:**

Baseline versus after-intervention median (IQR) of IL-6 (pg/ml), CRP (mg/L) and mean ± SD of ESR (mm) in garlic and placebo groups was 2.2 (0.8, 6.4) versus 0.7 (0.6, 1.2) (*p* <  0.001) and 2.0 (0.8, 2.1) versus 0.6 (0.6, 0.8) (*p* = 0.002), 13.0 (5.0, 14.0) versus 2.0 (1.0, 9.0) (p <  0.001) and 7.0 (2.0, 10.0) versus 6.0 (3.7, 7.5) (*p* = 0.547) and 35.4 ± 21.7 versus 50.7 ± 28.5 (*p* = 0.021) and 46.0 ± 26.0 versus 45.3 ± 22.3 (*p* = 0.797). Median (IQR) of Percentage Before-After change in CRP was − 71.4%(− 85.7, − 42.9%) and − 20.0%(− 30.0, 114.3%) in garlic and placebo group respectively. The Mann-Whitney U test indicated this difference is statistically significant (*p* <  0.001).

**Conclusion:**

The results imply that administrating 400 mg of standardized garlic extract twice a day for 8 weeks resulted in a significant reduction in IL-6, CRP and ESR. Since inflammatory state can be a serious life threatening condition in PD patients, we suggest prescribing this safe and well-tolerated natural substance to attenuate the inflammatory state in these patients. However, assessment of these effects in a larger randomized trial is strongly recommended (IRCTID: IRCT2017072535305N1, 2017-10-16).

## Background

End-stage renal disease (ESRD) patients experience worse outcomes in comparison to general population due to dependency of their life prolongation on maintenance dialysis [1]. The mortality of dialysis patients is 6.1 to 7.8 times greater than age-matched general population [2]. Cardiovascular disease (CVD) is considered as main cause of morbidity and mortality in ESRD patients. However, traditional risk factors of CVD are only partially responsible for high cardiovascular burden in these patients. Inflammation remains as a nontraditional but critical risk factor to cause CVD in patients with chronic kidney disease [3]. The prevalence of systemic inflammation is estimated between 12 and 65% in peritoneal dialysis (PD) patients [4] who account for 11% of the total dialysis population worldwide [5]. According to a review study, C-reactive protein (CRP) and interluekin 6 (IL-6) as the most common inflammatory markers in PD patients were associated with mortality and cardiovascular outcomes in PD and hemodialysis patients [2].

Utilization of herbal and plant foods in prevention of diseases is noticeably grown in recent years [6]. Garlic (*Allium sativum*) as a well-known useful food substance is one of these plants. Utilization of garlic as an effective vegetable against various disorders has been approved for centuries [7]. Several studies confirmed the effectiveness of garlic in lowering of blood lipids [8–10], glucose [9, 11], systolic blood pressure [10, 12, 13] and inflammatory biomarkers [6, 14, 15]. Some of mechanisms for potential anti-inflammatory effects of this substance has been attributed to decrease cytokine production in endothelial cells [16], creating an anti-inflammatory gene expression profile and modify adipocyte metabolic profile [15]. To the best knowledge, there is no study which has assessed the effects of garlic on inflammatory and other markers in PD patients. Hence, examining the effects of garlic on inflammation as one of the most important risk factors for CVD in PD patients (that is also the major cause of their mortality and morbidity), can be highly warranted. This study was aimed to investigate the effects of garlic on such markers of inflammation, lipid profile, liver, renal, and peritoneal function test plus some other biomarkers in PD patients.

## Methods

### Subjects and study design

**Fig. 1:**
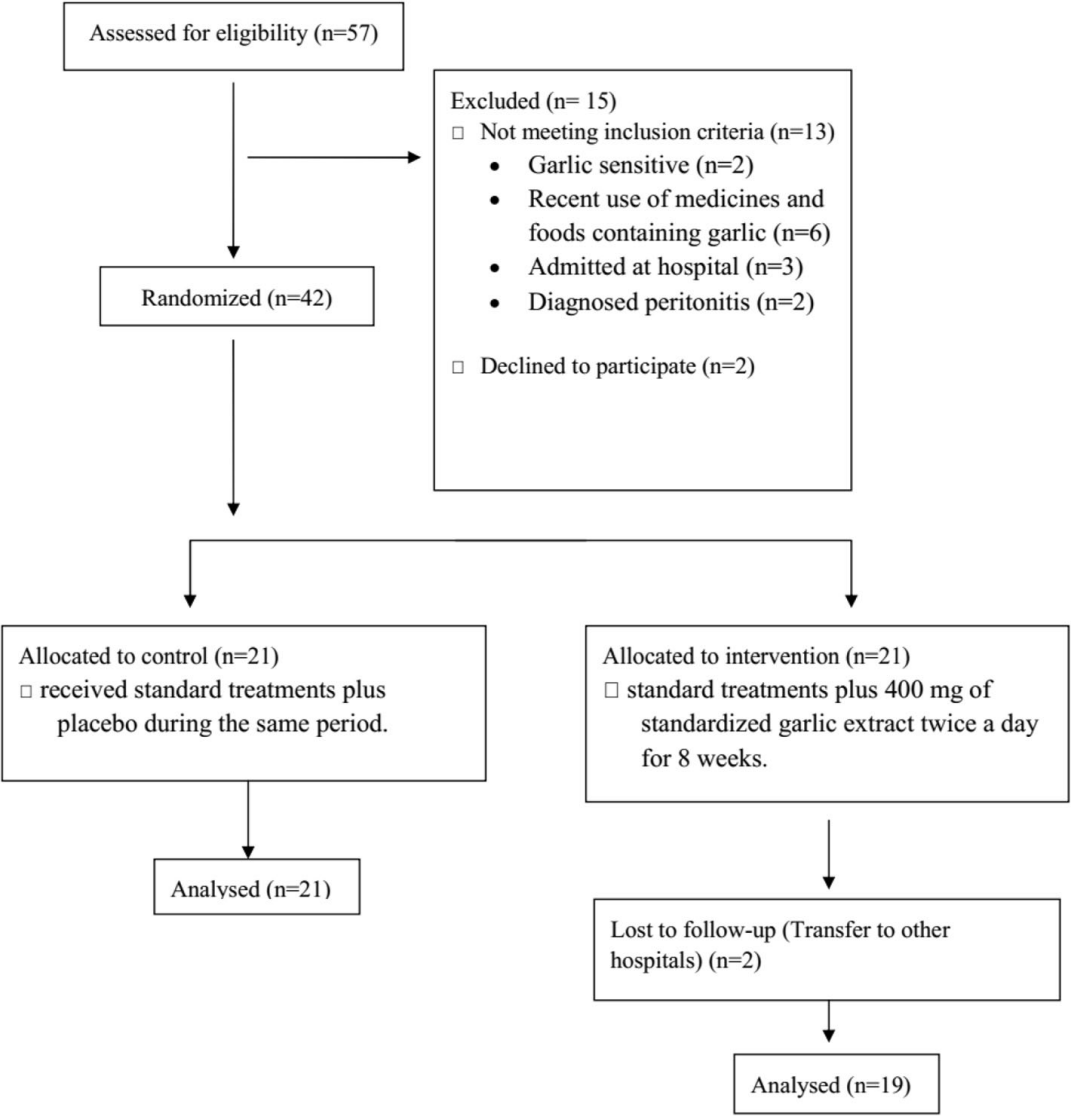
Flowchart of the study

In this parallel-designed double blind randomized clinical trial (ratio 1:1), 42 PD patients at the dialysis center of Shafa dialysis center, Tehran in 2017 were included. Subjects were eligible if they were PD patients between the age range of 18 to 80, garlic insensitive and no recent use of medicines and foods containing garlic. The patients who had a history of hemorrhagic disorders or were taking medications like warfarin, had diagnosed digestive disease such as ileal bypass and alcohol consumption were excluded from the study (Fig. 1).

In the current study, patients were randomly allocated into two groups of intervention and conventional treatment by balanced block randomization technique with 4 blocks. STATA software generated random numbers chains 1 to 6 until the desired sample size was achieved. Given that the total number of cases to fit two people in 4-blocks is 6 modes, if the generated number exceeded 6, the next number was regenerated, regardless of the previous number. Preparation of sequences of random allocation of cases and putting them in sealed envelopes (airtight) and numbered with a five-digit serial number were performed by a third person who was not involved in the study design. All envelopes (*n* = 40) had a random 5-digit serial number that was opened immediately after completion of the basic information and examinations of participants and participants were allocated into the intervention group (Garlic extract) or control group (Current treatment plus the garlic extract placebo).

Intervention group received standard treatments plus 400 mg of standardized garlic extract twice a day for the time period of 8 weeks while control group received standard treatments plus placebo during the same period. Garlic extract was prepared in the form of tablets containing 1 mg (1000 mcg) of Alliin. Placebo and garlic tablets were completely matched in appearance and they were both manufactured by the same pharmaceutical company (Amin chemical & pharmaceutical company).

The primary outcome in this study was systemic inflammation which was evaluated by measuring the concentrations of IL-6 and CRP and Erythrocyte sedimentation rate (ESR) in serum. The secondary outcomes were lipid profile (total cholesterol and triglyceride (TG)), liver function tests (serum glutamate pyruvate transaminase (SGPT) and serum glutamic oxaloacteic transaminase (SOGT)), renal function tests (renal Kt/V; urine volume), normalized protein catabolic rate (nPCR) and albumin concentration, peritoneal function tests (peritoneal Kt/V; and peritoneal ultrafiltration volume), and some other biomarkers including homocystein, sodium, potassium, phosphorus, calcium, ferritin, uric acid and parathormon. These outcomes were measured twice for each patient: once before the administration of interventions and once after the 8-week treatment period. The written informed consent was obtained from all individual participants included in the study. The proposal of this research has been approved in the Ethics Committee of Shahid Beheshti University of Medical Sciences (IR.SBMU.MSP.REC.1396.114).

### Sample size

Since this is the first study about effects of garlic on inflammatory markers in peritoneal dialysis patients, according to the optimal sample size estimation for a pilot randomized trial approach [17], a sample size of 40 participants (20 in each of the intervention and control groups) will be sufficient to detect a clinically important effect size of 35% (small to medium effect size) between groups, using a two-sided Z-test of the difference between proportions with 90% power and a 5% significance level.

### Biochemical assays and laboratory measurements

Venous Blood Samples obtained from each participant after an overnight fast,before and 8 weeks after garlic extract tablets or placebo intake. Samples collected into standard simple plain vacutainer tubes and allowed to stand at room temperature for 20 min to clot, then centrifuged for 10 min.Serum aliquots prepared for storage at − 20 C until future analysis in the same assay.

Biochemical parameters done (Total Cholesterol,Triglyceride,Creatinine,Urea,Alanine Aminotransferase, Aspartate Aminotransferase) on blood samples that collected in serum clot activator tubes then assayed in standard automated analyzer using commercial kits with standard methods. Samples for hemoglobin concentration collected into tubes containing EDTA at screening and after taking the tablets. Homocysteine concentrations measured by using human Homocysteine (HCY) kits (Siemens Healthcare Diagnostics, Germany) by biochemical and quantitative luminance methods in serum collected samples.

Measurement of IL-6 levels in serum determined using an ELISA (Human Enzyme-linked immunosorbent assay) kits (IBL Immuno_Bioligical Labratories,International GmbH,Hamburg, Germany). level of high-sensitivity C-reactive protein (hsCRP) studied with use of latex turbidimetric immunoassay method with a hsCRP kit on the Behring Nephelometer 100 Analyzer and the results expressed as mg/L.

Samples measured in duplicate manufacturer recommended wave length against a known standard curve depending on the specifications of the protocol.

### Statistical analysis

Departure from normality assumption was assessed by the Kolmogorov-Smirnov test. Mean ± standard deviation (SD) or median (inter-quintile range) was used for presenting the data with normal or non-normal distribution, respectively. For variables with normal distribution including ESR; total cholesterol; calcium; phosphorus; potassium; albumin; uric acid; peritoneal Kt/v; ultrafiltration; ferritin and nPCR, we assessed the effect of intervention via analysis of covariance (ANCOVA) with treatment as fixed effects and with baseline values and age as covariates. For other variables, we calculated the difference of outcomes values from baseline to the end of the 8-week treatment period for each person and compared it between the two groups using Mann-Whitney test. As an ancillary analysis, we compared baseline values of biochemical markers with their post-treatment values in each group separately by paired t-test or Wilcoxon signed rank test. A *p*-value below 0.05 was considered significant in all analyses. All statistical analyses were performed using the SPSS software version 18.

## Results

The recorded data of 40 PD patients (19 in intervention group, 21 in control group) were analyzed. Mean ± SD of age was 52.8 ± 18.2 and 56.0 ± 16.1 in garlic-treated and placebo-treated group, respectively. The baseline biochemical characteristics of the two groups are presented in Table 1. As shown, two groups were similar by most of biochemical characteristics at baseline.

**Table 1:** Baseline Biochemical Characteristics of Patients Receiving Garlic powder or Placebo

Variable		Control group (N = 21)	Garlic group (*N* = 19)	*P*-value
	Age (Year)	52.8 ± 18.8	56.0 ± 16.1	0.56^a^
	BMI (Kg/m^2^)	25.9 ± 4.5	26.3 ± 5.4	0.80^a^
	Peritoneal dialysis duration (month)	30.7 ± 3.2	33.1 ± 2.8	0.47^a^
Gender	Male	9 (42.8)	8 (42.1)	0.88^b^
	Female	12 (57.2)	11 (57.9)	
Inflammatory markers	IL-6 (pg/ml)	2.0 (0.8, 2.1)	2.2 (0.8, 6.4)	0.169^c^
	CRP (mg/l)	7.0 (2.0, 10.0)	13.0 (5.0, 14.0)	0.014^c^
	ESR (mm)	46.0 ± 26.0	50.7 ± 28.5	0.586^a^
Lipid profile	Cholesterol (mg/dl)	167.7 ± 38.4	206.8 ± 61.4	0.024^a^
	Triglyceride (mg/dl)	170.0 (112.5184.0)	134.0 (103.0, 195.0)	0.989^c^
Liver function	SGPT (IU/l)	16.0 (12.0, 22.0)	16.0 (15.0, 26.0)	0.216^c^
	SGOT (IU/l)	17.0 (12.5, 22.0)	20.0 (13.0, 38.0)	0.278^c^
Renal function	Renal Kt/V	0.3 (0.0, 1.1)	0.3 (0.1, 1.3)	0.592^c^
	Urine volume (ml)	300.0 (0.0,1400.0)	500.0 (200.0,1000.0)	0.670^c^
	nPCR	0.7 ± 0.2	0.8 ± 0.2	0.385^a^
	Albumin (gr/dl)	4.3 ± 0.7	4.2 ± 0.5	0.515^a^
Peritoneal function	Peritoneal KTV	1.4 ± 0.4	1.5 ± 0.4	0.836^a^
	Ultrafiltration (ml)	830.4 ± 495.9	887.9 ± 620.8	0.747^a^
	Potassium (mEq/l)	4.3 ± 0.6	4.5 ± 0.6	0.295^a^
	Phosphor (mg/dl)	5.2 ± 0.7	4.9 ± 1.1	0.245^a^
	Calcium (mg/dl)	9.8 ± 0.7	9.7 ± 1.1	0.582^a^
	Ferritin (ng/ml)	400.6 ± 332.5	319.8 ± 224.5	0.974^a^
	Uric acid (mg/dl)	6.5 ± 1.0	5.6 ± 1.3	0.017^a^
	Hemoglobin (g/dl)	11.4 (10.1–15.0)	11.7 (6.4–16.7)	0.97^c^
Other markers	Parathormon (pg/ml)	106.0 (49.0–171.0)	108.0 (70.0–138.0)	0.78^c^
	Homocysteine (mcmol/l)	17.5 (12.3, 30.1)	22.7 (18.2, 42.0)	0.076^c^
Drug History	Calcitriol	7 (41.2)	10 (58.8)	0.33^b^
	Statins	8 (34.8)	15 (65.2)	0.012^b^
	ACE Inh/ ARB	6 (28.6)	6 (31.6)	1.0^b^
	Furosemide	21 (100)	19 (100)	-^b^
	Aldactone	0 (0)	0 (0)	-^b^
	Beta blocker	1 (4.8)	0 (0)	1.0^b^

*IL-6* Interleukin 6, *CRP* C-reactive protein, *ESR* erythrocyte sedimentation rate, *SGPT* serum glutamic pyruvic transaminase, *SGOT* serum glutamic-oxaloacetic transaminase, and n-PCR: normalized protein catabolic rate

^a^values are presented as mean ± SD and compared by Independent T-test

^b^Values are presented as number (%) and compared by chi square test

^c^values are presented as median (IQR) and compared by Mann-Whitney Test

### Inflammatory markers

For inflammatory marker with normal distribution (ESR), we compared after-intervention concentrations between the two group using analysis of analysis of covariance ANCOVA. During 8 weeks of garlic treatment, the mean ESR significantly decreased (Table 2).

**Table 2:** Post-treatment comparison of biochemical characteristics with normal distribution in control or garlic group

Variable		Control group (*N* = 21)	Garlic group (*N* = 19)	Difference	*P*-value^a^
		Mean ± SD	Mean ± SD	Mean (95% CI)	
Inflammatory marker	ESR (mm)	45.3 ± 22.3	35.4 ± 21.7	9.9 (− 4.3, 24.0)	0.027
Lipid profile	Cholesterol (mg/dl)	153.6 ± 31.6	187.6 ± 36.9	−34.0 (− 55.9, − 12.1)	0.115
Renal function	nPCR	0.8 ± 0.2	0.8 ± 0.2	− 0.02 (− 0.1, 0.1)	0.849
	Albumin (g/dl)	4.2 ± 0.7	4.0 ± 0.5	0.2 (− 0.2, 0.6)	0.264
Peritoneal function	Peritoneal Kt/V	1.4 ± 0.5	1.4 ± 0.4	0.02 (− 0.3, 0.3)	0.511
	Ultrafiltration (ml)	1085.7 ± 589.3	973.2 ± 523.6	112.5 (− 245.8, 470.9)	0.104
	Potassium (mEq/l)	4.2 ± 0.5	4.7 ± 0.6	− 0.4 (− 0.8, − 0.1)	0.039
	Phosphor mg/dl)	5.3 ± 1.1	5.1 ± 1.3	0.2 (− 0.6, 0.9)	0.562
	Calcium (mg/dl)	10.0 ± 0.7	9.6 ± 0.7	0.3 (− 0.1, 0.8)	0.240
	Ferritin (ng/ml)	394.5 ± 381.9	280.3 ± 190.6	114.2 (− 82.3, 310.6)	0.339
	Uric acid (mg/dl)	6.5 ± 1.4	6.1 ± 1.4	0.5 (− 0.4, 1.4)	0.221

*ESR* erythrocyte sedimentation rate, *SGOT* serum glutamic-oxaloacetic transaminase, and n-PCR: normalized protein catabolic rate

^a^values are tested by analysis of covariance (ANCOVA)

For inflammatory markers with non-normal distribution (IL-6, CRP, and homocysteine), we calculated the change from baseline to the end of the 8-week treatment period for each patient and compared it between the groups. The results are given in Table 3. As shown, the occurred decrement in inflammatory biomarkers is more remarkable in garlic than placebo group. However, a significant and borderline significant difference between two groups was observed just for CRP and IL-6, respectively.

**Table 3:** Before-after changes of biochemical characteristics with non-normal distribution between the control and garlic group

Variable		Absolute Before-After change			Percentage of Before-After change		
		Control group (*N* = 21)	Garlic group (*N* = 19)	*P*-value ^a^	Control group (*N* = 21)	Garlic group (N = 19)	P-value
		Median (IQR)	Median (IQR)				
IM	IL-6 (pg/ml)	−0.6 (− 1.4, − 0.2)	−0.9 (− 5.2, − 0.2)	0.097	−39.7% ± 28.0%	−51.7% ± 28.3%	0.185 ^b^
	CRP (mg/l)	− 1.0 (− 3.0, 2.0)	−4.0 (− 11.0, − 3.0)	< 0.001	−20.0% (− 30.0, 114.3%)	− 71.4% (− 85.7, -42.9%)	< 0.001 ^c^
LP	Triglyceride (mg/dl)	−7.0 (− 38.0, 38.0)	− 9.0 (− 58.0, 10.0)	0.408	−4.1% (− 20.6, 36.9%)	− 5.7% (− 21.8, 14.3%)	0.238 ^c^
LF	SGPT (iu/l)	0.0 (− 7.0, 3.0)	−2.0 (− 10, 4.0)	0.688	1.2% ± 40.3%	−6.2% ± 37.5%	0.553 ^b^
	SGOT (iu/l)	−1.0 (− 4.0, 2.0)	−1.0 (− 10.0, 1.0)	0.586	−4.9% ± 24.9%	−8.8% ± 41.9%	0.721 ^b^
RF	Renal Kt/V	0.00 (− 0.04, 0.05)	0.01 (− 0.03, 0.17)	0.467	2.3% (− 16.3%, 38.9%)	4.0% (− 13.6%, 45.5%)	0.661 ^c^
	Urine volume (ml)	0.0 (0.0, 400.0)	0.0 (0.0, 200.0)	0.411	58.8% (− 12.7, 100.0%)	10.0% (− 28.6%, 54.5%)	0.288 ^c^
OM	Parathormon (pg/ml)	29.0 (− 9.5, 85.5)	23.0 (− 11.0, 86.0)	0.946	50.8% (− 11.0%, 83.0%)	32.9% (− 15.9, 90.0%)	0.903 ^c^
	Homocysteine (mcmol/l)	−4.7 (− 10.5, −1.9)	− 6.7 (− 18.2, − 5.5)	0.129	−27.3% (− 46.0, − 10.8%)	−35.1% (− 45.4, −29.5%)	0.323 ^c^

*IM* Inflammatory Markers, *LP* Lipid Profile, *LF* Liver Function, *RF* Renal Function, *OM* Other Markers, *IL-6* Interleukin 6, *CRP* C-Reactive Protein, *SGPT* Serum Glutamic Pyruvic Transaminase

^a^values are tested by Mann-Whitney U test

^b^values are presented as mean ± SD and compared by Independent T-test

^c^values are presented as median (IQR) and compared by Mann-Whitney U test

As an ancillary analysis, we compared baseline values of these markers with their post-treatment values in each group separately by paired t-test or Wilcoxon signed rank test, in appropriate to normality assumption. According to this analysis, in garlic group, IL-6, CRP and ESR were all significantly reduced while in placebo group, a significant decrement just for IL-6 was observed (Table 4). The magnitude of occurred changes in these markers was noticeably greater in garlic group.

### Other markers

Among other markers that were assessed we observed a significant difference between the two groups just for potassium (Table 2). Garlic significantly increased serum potassium levels. In within group comparisons, there was a significant increment just for uric acid and parathormon and a significant decrement just for homocysteine in garlic group (Table 4). The significant changes in control group included a decreasing in cholesterol and homocysteine and an increasing in ultrafiltration (Table 4).

**Table 4:** Within Group Comparison of Biochemical Characteristics of Patients in Control or Garlic group

Variable		Control group (*N* = 21)			Garlic group (*N* = 19)		
		Before	After	P-value	Before	After	P-value
IM	IL-6 (pg/ml)	2.0 (0.8, 2.1)	0.6 (0.6, 0.8)	0.002^a^	2.2 (0.8, 6.4)	0.7 (0.6, 1.2)	< 0.001^a^
	CRP(mg/l)	7.0 (2.0, 10.0)	6.0 (3.7, 7.5)	0.547^a^	13.0 (5.0, 14.0)	2.0 (1.0, 9.0)	< 0.001^a^
	ESR (mm)	46.0 ± 26.0	45.3 ± 22.3	0.797^b^	50.7 ± 28.5	35.4 ± 21.7	0.021^b^
LP	Cholesterol (mg/dl)	167.7 ± 38.4	153.6 ± 31.6	0.048^b^	206.8 ± 61.4	187.6 ± 36.9	0.053^b^
	Triglyceride (mg/dl)	170.0 (112.5, 184.0)	146.0 (131.5, 186.5)	0.601^a^	134.0 (103, 195.0)	137.0 (90.0, 200.0)	0.334^a^
LF	SGPT (iu/l)	16.0 (12.0, 22.0)	16.0 (10.0, 21.0)	0.342 ^a^	16.0 (15.0, 26.0)	19.0 (15.0, 22.0)	0.197 ^a^
	SGOT (iu/l)	17.0 (12.5, 22.0)	16.0 (11.0, 22.0)	0.422^a^	20.0 (13.0, 38.0)	20.0 (13.0, 22.0)	0.120 ^a^
RF	Renal Kt/V	0.3 (0.0, 1.1)	0.3 (0.0–2.2)	0.599^a^	0.3 (0.1, 1.3)	0.3 (0.0–4.6)	0.155^a^
	Urine volume (ml)	300.0 (0.0, 1400.0)	500.0 (0.0, 1550.0)	0.141^a^	500.0 (200.0, 1000.0)	500.0 (200.0, 1000.0)	0.267^a^
	nPCR	0.7 ± 0.2	0.8 ± 0.2	0.423^b^	0.8 ± 0.2	0.8 ± 0.2	0.861^b^
	Albumin	4.3 ± 0.7	4.2 ± 0.7	0.285^b^	4.2 ± 0.5	4.0 ± 0.5	0.051^b^
PF	Peritoneal KTV	1.4 ± 0.4	1.4 ± 0.5	0.438^b^	1.5 ± 0.4	1.4 ± 0.4	0.136^b^
	Ultrafiltration (ml)	830.4 ± 495.9	1085.7 ± 589.3	0.004^b^	887.9 ± 620.8	973.2 ± 523.6	0.398^b^
	Potassium (mEq/l)	4.3 ± 0.6	4.2 ± 0.5	0.920^b^	4.5 ± 0.6	4.7 ± 0.6	0.184^b^
	Phosphor (mg/dl)	5.2 ± 0.7	5.3 ± 1.1	0.560^b^	4.9 ± 1.1	5.1 ± 1.3	0.179^b^
	Calcium (mg/dl)	9.8 ± 0.7	10.0 ± 0.7	0.426^b^	9.7 ± 1.1	9.6 ± 0.7	0.871^b^
	Ferritin(ng/ml)	400.6 ± 332.5	394.5 ± 381.9	0.805^b^	319.8 ± 224.5	280.3 ± 190.6	0.224^b^
	Uric acid (mg/dl)	6.5 ± 1.0	6.5 ± 1.4	0.733^b^	5.6 ± 1.3	6.1 ± 1.4	0.035^b^
OM	Parathormon (pg/ml)	106.0 (49.0, 171.0)	180.0 (77.5, 209.0)	0.092	108.0 (70.0, 138.0)	130.0 (85.0, 230.0)	0.018
	Homocysteine (mcmol/l)	17.5 (12.3, 30.1)	12.0 (9.0, 21.3)	0.035	22.7 (18.2, 42.0)	16.0 (10.8, 25.0)	< 0.001

*IM* Inflammatory Markers, *LP* Lipid Profile, *LF* Liver Function, *RF* Renal Function, *PF* Peritoneal Function, *IL-6* Interleukin 6, *CRP* C-Reactive Protein, *ESR* Erythrocyte Sedimentation Rate, *SGPT* Serum Glutamic Pyruvic Transaminase, *SGOT* Serum Glutamic-Oxaloacetic Transaminase; and n-PCR: normalized Protein Catabolic Rate

^a^values are presented as median (IQR) and compared by Wilcoxon Test

^b^values are presented as mean ± SD and compared by Paired T-test

## Discussion

This was a double-blind placebo-controlled trial that investigated the effect of garlic on some of inflammatory markers as primary outcomes and lipids profile, liver and renal function, peritoneal function and some other biomarkers as secondary outcomes.

As the main finding, this study showed that garlic can be effective in reducing the inflammatory biomarkers in ESRD patients. We observed that the levels of IL-6, CRP and ESR significantly decreased after the end of 8-week period in garlic-treated group. Similar to our study, Mozaffari-Khosravi et al. [18] investigated the effect of garlic on pro-inflammatory markers in 44 postmenopausal osteoporotic women and did not observe a significant difference in inflammatory cytokines of IL-1, IL-6 and tumor necrosis factor alpha (TNF-α) between garlic and placebo groups. They saw a significant reduction in TNF-α just in garlic group after the intervention [18]. A small sample size may be a cause for insignificant results in both studies. According to a systematic review, garlic can reduce inflammatory markers, including CRP and TNF-α. This study concluded that high concentration CRP increases the odds of coronary artery disease (OR = 1.45; 95% CI: 1.25–1.68) and that garlic consumption can improve cardiovascular health via lowering pro-inflammatory cytokines [19]. In an in-vitro study, a significant reduction was seen in several inflammatory biomarkers including tumor TNF-α, IL-1α, IL-6, IL-8, T-cell interferon-gamma (IFN-γ), and IL-2, in the presence of ≥10 μg/ml garlic extract in inflammatory bowel disease [20]. Ghodsi et al. observed that administration of garlic extract along with regular swimming trainings for 8 weeks was effective in reducing age dependent renal tissue inflammation in normal rats [21].

So, garlic can be considered as a useful natural herb in inhibition of inflammation. The advantageous effects of garlic on health are due to organosulfur compounds in it [7]. Ban et al. indicated that thiacremonone, a sulfur compound from garlic, prohibits Nuclear Factor Kappa B NF-κB activation through interacting with sulfhydryl group of nuclear factor Kappa B (NF-κB) molecules [22]. Lee et al. identified some sulfur-containing compounds from garlic including Z- and E-ajoene and oxidized sulfonyl derivatives of ajoene that inhibit the expression of pro-inflammatory cytokines such as tumor necrosis factor-α, IL-1β, and IL-6 [23]. Considering the fact that elevated values of pro-inflammatory cytokines are associated with greater odds of developing coronary artery disease [19] and therefore, an independent predictor of mortality in ESRD patients [3], we suggest to administrate garlic in these patients as a beneficial remedy.

About secondary outcomes, we observed a significant increment in serum potassium level in garlic treated group than control group. A similar finding was also reported by Oluwole [24] in studying the effects of garlic on some biochemical parameters. He concluded that garlic can help in maintenance of electrolyte balance. According to this claim, enhancing sodium and potassium reabsorption following garlic administration can improve renal function. In addition, he believed that the ameliorating effect of garlic on hypertension can be partly explained by this mechanism, i.e., increasing renal reabsorption of basic electrolytes such as sodium and potassium due to probable incremental effect of garlic on renal blood flow may secondarily relieve the hypertension. Meanwhile, Pedraza-Chaverri et al. [25] indicated that garlic is effective in improving renal injury induced by potassium dichromate (K2Cr2O7) due to its antioxidant properties.

Other checked secondary outcomes were lipid profile, liver; renal and peritoneal function, parathormon and homocysteine. We observed a significant reduction in cholesterol concentration within both groups. Majority of this reduction was higher in garlic treated group than placebo treated group (− 9.30% versus − 8.4%, respectively). However, similar to Doorn et al. [16] we didn’t observe a significant difference between two groups. Contrary to our study, the beneficial effect of garlic on lipid profile parameter is indicated in many meta analyses [10, 26, 27]. According to Cicero et al. [28] the most important molecule in garlic with lipid-lowering effect is allicin that acts via multiple mechanisms including inhibiting synthetase enzymes including HMG-CoA reductase, squalene-monooxygenase, and acetyl-CoA; reducing cholesterol endogenous synthesis due to directly reacting with nonacetylated-CoA; blocking the dietary cholesterol absorption and increasing excretion of bile acids. These can be the important findings considering the increase of lipid profile represents a traditional and critical risk factor of CVD in patients with chronic kidney disease. Shabani et al. [29] concluded to prescript of this safe and tolerable herb specially for patients with mild hyperlipidemia and chemical drug intolerance.

Uric acid concentration was significantly increased after the 8-weak intervention in garlic group. This finding is inconsistent to other studies [30, 31]. Parathormon concentration was significantly increased after the 8-weak intervention in garlic group. There was no statistically significant difference in homocysteine concentration between the two groups after the intervention. This is similar to findings in a clinical trial on patients with ischemic heart disease [32]. However, we witnessed a significant decrease in its concentration within both groups. Other assessed biomarkers didn’t show a significant change after the intervention too.

As a limitation, it should be mentioned that the small size of the investigated samples might be insufficient to indicate the effects of garlic on various biomarkers. This obliged us to use nonparametric tests with lower power to detect potential differences between the two groups. Hence, we recommend conducting more trials with large sample sizes in order to investigate the potential beneficial effects of garlic in PD patients more precisely.

In conclusion, oral treatment with 400 mg of standardized garlic extract twice a day and for 8 weeks resulted in a significant reduction in CRP and ESR.

## Conclusion

Regarding the fact that high concentration of these inflammatory markers can be a serious life threat for peritoneal dialysis patients, we suggest that this safe and well-tolerated natural substance be prescribed in an attempt to attenuate the inflammatory response in these patients. However, the assessment of these effects in larger trials is strongly recommended.

## Abbreviations

ANCOVA: Analysis of covariance
ANCOVA: Analysis of covariance
CRP: C-reactive protein
CVD: Cardiovascular disease
ESR: Erythrocyte sedimentation rate
ESRD: End-stage renal disease
HCY: Human Homocysteine (HCY)
IFN-γ: Interferon-gamma
IL-6: Interluekin-6
IQR: Inter quartile range
IQR: Interquartile range
NF-κB: Nuclear factor Kappa B
nPCR: normalized protein catabolic rate
PD: Peritoneal dialysis
SD: Standard deviation
SGOT: Serum glutamic oxaloacteic transaminase
SGPT: Serum glutamate pyruvate transaminase
TG: Triglyceride
TNF-α: tumor necrosis factor alpha ()
